# Consumption

**Published:** 1882-04

**Authors:** 


### CONSUMPTION

Is a disease which destroys one out of every six grown persons, which invades every family in the civilized world, which carries to the grave, from New York city alone, in the space of a single year, more than three thousand persons. Such a disease ought to be understood, at least as to its general features. Every intelligent individual ought to have a general idea of its nature, its causes, and its prevention. Such a result will be of slow attainment. The thoughtful few will give it attention, which will gradually force itself on the masses, at least so far as the general principles of prevention are concerned.

We believe that, within a few years, there has been a progressive popular intelligence on the subject, and that more correct views prevail in reference to its nature, than at any previous time. Now and then a man of extraordinary intelligence and practical good sense makes his appearance, such as Moreton, of Philadelphia, Wooster, of Cincinnati, and Norcom, of Carolina; but they have passed away, and in a whole lifetime succeeded in impressing their views upon a chance individual here and there.

Two sentiments pervaded the theory and practice of these excellent men—and they were all contemporaries, but most probably wholly unknown to each other. Moreton was widely known; we never saw the amiable Wooster's name in any printed book; and we ourselves never heard of Norcom until years after his death. They believed that—

1st. No medicine known to them had any uniform appreciable good effect in common consumption of the lungs.

2d. Without a free exposure to out-door air, no case of consumption ever was cured.

These same sentiments are now taking hold of the medical minds of Great Britain, with several remarkable additions: that—

1st. Sea voyages hasten the fatal result of consumptive disease.

2d. Seeking a milder climate precipitates a like result.

3d. The maintenance of an equal artificial temperature only aggravates the disease.

"There is an antecedent state of disordered health, which, as a causative agent, originates that altered state of the blood which produces tubercle. To this part of our subject, I would entreat your earnest attention. It is not only the key to the disease, but it is the hopeful period for treatment—the critical time in which we may check the inroads of the most fatal of all affections incident to the human frame. Feel no hesitation in saying that the earliest symptom of consumption is 'WASTING;' it precedes cough, and spitting of blood, and hectic, and all the physical signs; * * * hence the second symptom is *debility*."

The diseases to which the lungs and their proper appendages are liable, are Asthma, Bronchitis, Consumption, Laryngitis or Throat ail, Croup, Pleurisy, Inflammation of the Lungs, Congestion of the Lungs, Quinsy, &c. All these diseases arise from two causes:

1. Changes of temperature.
2. The failure to keep the breathing apparatus in vigorous, full, healthful operation, by a sufficient amount daily of exertive exercise in the open air. By a wise attention to this second cause of lung diseases, the former will cease to be a cause, except in occasional cases. Not only so, threatened consumption may be effectually warded off, in the vast majority of cases, by the proper adaptation of daily out-door exertive exercise to the requirements of the system, as indicated by the condition of the pulse, the heart, the breathing organs, of which the physician ought to be the standing judge. For, when a man is an invalid, the amount of food, air, and exercise which he needs, requires as much of medical intelligence, experience, and skill, as would the judicious exhibition of medicine.

*Deficiency in breath, and strength, and flesh*, are the first far-off symptoms of consumption, coming on as they do long before cough, and spitting of blood, and pains about the chest. We believe that, in the vast majority of cases, the foundation—the first symptoms of consumption in both sexes, is laid at about the age of eighteen or twenty years, and that, if medical attention were secured at this age to children, by which a critical examination could be made as to those points, and corresponding action follow, the mortality from this disease would be largely diminished at once. To neglect these earliest symptoms of consumption, is like neglecting looseness of bowels in Asiatic cholera, which is nothing short of almost certain death.

No one seems to have the slightest fear of consumption now-a-days, unless there is some cough, when the fact is, some persons have observed no cough at all until within three weeks of death, and on examination the lungs have been found a mass of rottenness. In the vast mass of cases, cough is a telegraphic signal that the train has been fired, that tubercles have been deposited, and are taking on irritative and inflammatory action, which is the stage immediately preceding actual decay of the lungs; whereas, if short breath, wasting of flesh, and general weakness, were considered, as they really are, the *avant couriers* of the disease, and corresponding action had, three fourths of those who now die of consumption could ward off the disease indefinitely. Hard-earned thousands, yes millions, often go to distant relations, as heartless as they are greedy, which else would have fallen to natural heirs, if, instead of nursing children from seventeen to twenty years, the moment they seem to become "*weakly*," by consigning them to feather beds, and furnaced rooms, and closed carriages, afraid as death of a single puff of fresh out-door air, lest it should give them a cold—we say a far different result would have followed the hard mattress, the cold sleeping-room, the frequent walk and daily horseback ride, early and late, of from five to thirty miles a day, in spite of all obstacles of wind, and storm, and dust, and snow, and hail—of zero or of broiling heat.

### *An example*—HEART DISEASE.

It is well known that the symptoms of a disease of the heart, and those of the lungs, as well as those of a spinal affection, are so apparently alike in the main, that it requires large medical experience to decide safely and certainly between them; but the exercise requisite in an affection of the lungs would inevitably destroy life if advised for a disease of the heart or spine. In no form of sickness is exercise so immediately and certainly fatal as in heart affections, while the results of active exercise in spinal disease are terrible, literally terrible, not in their immediate effects as involving life, but in the certain penalty of weeks, and months, and weary years of corporeal helplessness, and agonizing, almost ceaseless pain, requiring a thousand times more endurance and a far higher degree of fortitude than marching up to the cannon's mouth in the heat of battle. An affecting instance of this kind came under my notice within a few years past, and I feel sure that a recital of it will be a public benefit, as teaching the importance of taking early competent medical advice in cases of sickness.

I was called to see a young lady on a visit to New York, who was supposed to be in a decline. She was from a neighboring city, an only daughter. She was just entering life, with all the advantages which position, and fortune, and refinement, could bestow. She had a pulse of a hundred and twenty a minute, thirty-six respirations, an incessant cough, debility, such that she could not walk without two assistants. She still lives, a noble monument of heroic endurance and mental energy and worth. Previous to applying to me she had suffered a dozen deaths, in her efforts to take air and exercise on foot, on horse, in carriage; and as often almost as she would take them, she could, on reaching her own door, scarcely prevent herself from shrieking out with an agony of pain. She was encouraged to persevere in these efforts, and, with a daughter's affection for a loving mother, whose solicitude and watchfulness never slept, she did so, until locomotion became impossible. This being a clear case of spinal disease, every step she took, every moment she sat still, aggravated the complaint. The best medical skill in the country has failed to afford her any permanent relief.

### POUTING.

A little girl after playing awhile with some other children, suddenly broke up the happiness of the company by going home in a passion, because "they wouldn't play my way." This may be set down to mere waywardness in a child, but there is much of it in grown persons, in the great plays of practical life. There are persons of education, and culture, and of real benevolence, who refuse their co-operation in a good direction, simply because things are not done precisely in the way to suit themselves, and they go off to whine, and pout, and complain, pouring out there jeremiades in voluble profusion, or to waste their energies in crude experiments, or in impossible endeavors.

The "twelve," of olden time, while yet children in the knowledge of the kingdom, complained to the Master of another because "he followeth not us," is not of our "set," does not belong to our party, is not a member of our church, we are not acquainted with him, we know nothing about him. "Forbid him not" was the broad, and brotherly, and suggestive reply, embodying a generous principle of action for all time. Whoever does good, let him do it, bid him "God speed," "forbid him not."

Some men lack ballast, self-willed, self-confident, and with much apparent benevolence, are at the bottom ugly hearted. If a house is burning down, they will let it burn unless the fire is extinguished in precisely the way which suits them.

